# VEXAS Syndrome: A Comprehensive Review of Current Therapeutic Strategies and Emerging Treatments

**DOI:** 10.3390/jcm13226970

**Published:** 2024-11-19

**Authors:** Safi Alqatari, Abdulaziz A. Alqunais, Shahad M. Alali, Mohammed A. Alharbi, Manal Hasan, Mohammed D. Al Shubbar

**Affiliations:** 1 Rheumatology Division, Department of Internal Medicine, College of Medicine, Imam Abdulrahman Bin Faisal University, Dammam 31441, Saudi Arabia; sagqatari@iau.edu.sa (S.A.); mahasan@iau.edu.sa (M.H.); 2 College of Medicine, Imam Abdulrahman Bin Faisal University, Dammam 31441, Saudi Arabia; abdulazizalqunais@gmail.com (A.A.A.); Shahadma7711@gmail.com (S.M.A.);

**Keywords:** VEXAS syndrome, UBA1 mutation, autoinflammatory disorder, hematological disorders

## Abstract

VEXAS syndrome is a recently identified autoinflammatory disorder resulting from somatic mutations in the UBA1 gene, leading to a complex spectrum of severe inflammatory and hematologic manifestations. The absence of established treatment guidelines and the variability in clinical presentation make its management particularly challenging. Current therapeutic approaches are often based on limited evidence, and their effectiveness remains inconsistent. This review seeks to consolidate the existing knowledge on therapeutic strategies for VEXAS syndrome, offering a critical evaluation of their efficacy and addressing the gaps in the current literature. As the clinical recognition of VEXAS grows, there is an urgent need to explore more targeted, effective treatments that can address both the inflammatory and hematologic aspects of the disease. By providing a comprehensive analysis of the current therapeutic landscape, this review aims to guide clinicians and researchers toward developing more effective, long-term management strategies for this life-threatening condition.

## 1. Introduction

Vacuoles, E1 enzyme, X-linked, autoinflammatory, somatic (VEXAS) syndrome is a recently discovered somatic disorder characterized by severe autoinflammatory symptoms coupled with significant hematological abnormalities, primarily affecting adult populations. The syndrome first came to light through the examination of three patients who exhibited profound inflammatory responses and displayed overlapping clinical features, prompting the hypothesis of a potential common underlying genetic factor. In pursuit of this hypothesis, extensive exome sequencing was conducted on these initial patients. This detailed genetic analysis revealed a shared mutation in the UBA1 gene located on the X chromosome, establishing a direct genetic link to the syndrome. Building on this discovery, researchers expanded genetic screening to a larger cohort of patients with similar clinical profiles, including those with recurrent unexplained fevers, systemic inflammation, and other atypical, undiagnosed conditions. The expanded genetic analysis proved instrumental in identifying the mutation in approximately 25 additional patients, thereby broadening the clinical understanding of the disorder [1].

It is essential to recognize that the phenotypic spectrum of VEXAS syndrome remains incompletely defined and continues to expand as additional cases are identified. Patients commonly present with severe, treatment-resistant inflammatory and hematologic symptoms, which frequently follow a progressive and potentially fatal course [1,2,3]. Common manifestations of VEXAS syndrome include recurrent fevers, significant weight loss, relapsing polychondritis, neutrophilic dermatosis, leukocytoclastic vasculitis, neutrophilic alveolitis, and inflammatory arthritis. Additionally, rarer presentations involve gastrointestinal and peripheral nervous systems. Hematologic features are varied and include macrocytic anemia, thrombocytopenia, myelodysplastic syndrome, progressive bone marrow failure, multiple myeloma, monoclonal gammopathy of undetermined significance, and unprovoked thrombotic events, such as deep vein thrombosis and pulmonary embolism, which affect up to 50% of patients. Moreover, patients with VEXAS syndrome often present with complex symptoms that overlap with those of autoimmune or autoinflammatory conditions, such as Behçet’s disease and granulomatosis with polyangiitis, both of which commonly involve thrombotic complications [1,2,3,4,5]. It is also crucial to note that the expression of VEXAS syndrome can vary widely, largely influenced by the specific UBA1 mutation involved [2,6,7].

VEXAS syndrome is associated with substantial morbidity and mortality due to its severe autoinflammatory manifestations and complex hematologic complications. Without appropriate management, patients often face a progressively worsening and potentially fatal disease trajectory [1,3]. Prognosis is generally unfavorable, shaped by factors such as the intensity of systemic inflammation, the degree of hematologic involvement—including cytopenia and progression to myelodysplastic syndromes—and heightened risks of comorbid conditions like thrombosis and severe infections. Notably, prognosis varies among individuals and is influenced by the specific UBA1 mutation present [2,6,7], with certain mutations correlating with differing clinical outcomes. This variability highlights the critical need to understand the underlying pathophysiology, which will be discussed in the following section.

At present, there are no standardized treatment protocols or definitive clinical guidelines for the diagnosis and management of VEXAS syndrome, underscoring the intricate nature of this condition and the potential for under recognition or misdiagnosis in clinical practice. Currently, the literature reflects numerous efforts to address VEXAS syndrome, with each study contributing incremental insights while leaving critical gaps in understanding effective management strategies. In response to these uncertainties, this review aims to comprehensively examine all documented therapeutic interventions attempted for the management and treatment of VEXAS syndrome. Our approach entails a meticulous review of each medication and treatment strategy documented in the literature, covering both established and experimental therapies. Through this comprehensive analysis, we aim to consolidate the fragmented insights into a cohesive understanding of treatment efficacy and outcomes. This review seeks not only to clarify the current therapeutic landscape but also to highlight potential pathways for future research and clinical trials.

## 2. Pathophysiology

VEXAS syndrome presents with highly heterogeneous manifestations, exhibiting a mixed pattern of autoinflammation and autoimmunity. To optimize management and develop effective treatments for this disease, it is essential to attain a thorough understanding of its underlying pathophysiology. This deep insight is essential as it guides the creation of targeted therapies that specifically address the unique inflammatory and hematological characteristics of the syndrome. A comprehensive grasp of these mechanisms will not only enhance the effectiveness of current treatments but also create a path for innovative therapeutic strategies in the future.

### 2.1. The Ubiquitin–Proteasome System and UBA1 Gene

In every cell, a critical balance exists between protein synthesis and degradation, regulated by the ubiquitin–proteasome system (UPS). This system plays a crucial role in maintaining cellular health by preventing the accumulation of unnecessary or potentially harmful proteins, including those that are misfolded. For the proteasome to degrade proteins effectively and efficiently, these proteins must first be tagged with ubiquitin, through a process known as ubiquitination. The regulation of ubiquitination involves a cascade of three key enzymes: E1, the ubiquitin-activating enzyme; E2, the ubiquitin-conjugating enzyme; and E3, the ubiquitin ligase. Each plays a specific role in the tagging process, ensuring that proteins are correctly marked for degradation by the proteasome [8]. The roles of ubiquitin and UPS extend far beyond to play a crucial role in immune regulation, as any disruption to the UPS can lead to immune dysregulation and the inappropriate activation of immune pathways that are important in immune response and inflammation, leading to excessive inflammatory response [9]. The E1 enzyme, the initial enzyme in the ubiquitination cascade, is encoded by the UBA1 gene on the X chromosome [10].

Patients with VEXAS syndrome carry mutated forms of the UBA1 gene, primarily affecting the bone marrow. These mutations arise in pluripotent hematopoietic progenitors and, as these cells differentiate, persist exclusively in mature myeloid lineage cells, sparing other lineages [1]. Notably, initial studies focused on bone marrow, yet recent findings from a Spanish cohort have identified the UBA1 mutation in nail samples from nine individuals, though the implications of these findings remain unclear [11]. Going back to Beck et al.’s seminal study, three prevalent mutations within the UBA1 gene were identified—p.Met41Thr, p.Met41Val, and p.Met41Leu—among fifteen individuals [1]. These mutations are consistently reported across various studies [2,3,12,13,14], although they do not encompass the full genetic diversity, as atypical splice site mutations have also been observed [2,15,16,17,18].

The UBA1 gene encodes two primary isoforms, namely UBA1a and the cytoplasmic UBA1b, which begins transcription at codon 41 with the methionine initiation codon. The previously mentioned mutations frequently lead to an aberrant isoform, UBA1c, which is pathogenic and less functional, contributing to VEXAS pathology [1]. However, not all mutations result in UBA1c; for instance, Poulter et al. discovered a p.Ser56Phe substitution that decreases UBA1 activity without producing UBA1c, disrupting the ubiquitination process [16]. These mutations can give variable phenotypic manifestations. A French study involving 116 patients identified that the p.Met41Leu mutation is often associated with a milder clinical course, characterized by infrequent fever and less lung involvement, and is linked to a favorable prognosis with a 100% five-year survival rate. In contrast, the p.Met41Val mutation is associated with more severe systemic inflammation and hematological issues, as evidenced by higher C-reactive protein levels and increased myelodysplastic syndrome occurrences, though chondritis is less common in these patients, who generally have a poorer prognosis [2]. Another study highlighted the connection between specific mutations and dermatological manifestations, finding a strong correlation between the p.Met41Leu mutation and neutrophilic dermatosis, whereas the p.Met41Val mutation was frequently linked to vasculitic lesions, such as leukocytoclastic vasculitis, and mixed leukocytic infiltrates [7].

### 2.2. Inflammatory Profile

In a large prospective cohort of 40 VEXAS syndrome patients, Kosmider et al. delved into their immune responses, providing an in-depth analysis of the immunological behavior and molecular pathways in these patients. The study found that monocytes in VEXAS patients were not only diminished in number, due to increased cell death but also exhibited dysfunction and elevated expression of various chemokine receptors, specifically CXCR3 and CCR4, which explains skin involvement, and CXCR5 and CCR7, associated with the involvement of secondary lymphoid organs [19]. Increased cell death, particularly monocytes and neutrophils, was also demonstrated by a Japanese study, even after clinical remission, and this study suggests targeting cell death suppression as a potential management strategy [20].

Skin lesions were also analyzed, via RNAseq, revealing heightened gene expression of IL1B and IL6, along with the increased transcriptional activity of cytokines such as IL-1, IL-6, and TNF-alpha. Other cytokines like IFN-γ, IL-12B, IL-1β, IL-2, IL-23A, IL-5, and TNF were also noted. Notably, there was a marked upregulation of CXCR3 and CCR7. Taken together, these results underscore the crucial role of monocytes in the pathogenesis of VEXAS syndrome, where they are not only reduced but also functionally impaired, with enhanced chemokine receptor expression facilitating their migration to target organs and exacerbating local inflammation [19].

Concurrently, plasma analysis was conducted to delineate the inflammatory landscape in VEXAS patients, revealing a distinct ‘discriminating VEXAS signature’. This signature included a suite of positively correlated inflammatory mediators: IL-1α, IL-1β, IL-18, TGF-α, IL-7, galectin-3, and calprotectin. Additionally, two further inflammatory clusters were characterized within the VEXAS cohort. The first cluster was predominantly made up of inflammatory cytokines and proinflammatory chemokines such as IL-6, IL-1RA, CCL2/MCP-1, CCL11/eotaxin, CCL4/MIP-1β, CXCL2, RANTES, PDGF-AB, and EGF. The second cluster consisted largely of proinflammatory chemokines and growth factors, including CCL20/MIP-3α, CCL19/MIP-3β, CX3CL1, IL-10, G-CSF, GM-CSF, and VEGF. Significantly, IL-6 levels were notably elevated in VEXAS patients, emphasizing its role in intensifying inflammatory responses, while TNF-α and IFN-γ levels remained relatively unchanged. Furthermore, substantial increases were observed in IL-1β and IL-18, both crucial proinflammatory cytokines regulated by the inflammasome. These increases were supported by higher levels of IL-1RA, suggesting a dynamic counter-regulation of IL-1 activity in these patients. These observations collectively underscore a significant inflammatory dysregulation in VEXAS, with a particular emphasis on the activation of inflammasome-dependent pathways [19]. Moreover, another study highlights a similar pattern with the significant upregulation of genes related in various inflammatory pathways [21]. Nevertheless, these findings might not be consistently observed across different populations, as illustrated by a Spanish cohort study conducted by Mascaro et al., which failed to pinpoint any definitive inflammatory mediators in the disease pathogenesis, even when analyzing serum samples during periods of heightened disease activity [11]. It is important to note, however, that our understanding of the pathophysiology and inflammatory landscape of VEXAS syndrome is still evolving, and it may be too early to draw comprehensive conclusions.

## 3. Management

### 3.1. Steroids

Despite the complexity of VEXAS syndrome and its relative resistance to multiple therapeutic agents, systemic corticosteroids remain a standard and effective therapy, albeit not curative [22]. One of the two primary strategies for managing VEXAS focuses on inhibiting inflammatory signaling pathways and cytokines, a goal achieved through corticosteroid use [23]. Systemic corticosteroids are frequently administered empirically before the identification of the underlying UBA1 mutation and are considered a first-line treatment for managing the inflammatory symptoms and cytopenia associated with VEXAS syndrome [24,25].

Research has shown that high doses of corticosteroids (20 mg/day or more) can alleviate systemic inflammatory symptoms; however, attempts to taper the dosage often result in inadequate symptom control and recurrence [4,26,27,28,29]. Conversely, the long-term administration of high-dose corticosteroids may lead to steroid dependence and adverse complications, including cardiovascular events and infections, rendering corticosteroids an unfavorable option for prolonged use [28]. In a case reported by Alhomida et al., a patient with predominant dermatologic symptoms, fever, and anemia achieved complete symptom resolution using only oral prednisone, despite resistance to all steroid-sparing agents [27]. Additionally, a Spanish cohort study by Mascaró et al., involving 30 patients, demonstrated that high-dose corticosteroid therapy was the only intervention that effectively reduced inflammatory manifestations in all participants, with 70% achieving a complete response. However, this treatment showed limited efficacy for the hematologic abnormalities observed in these patients [11].

While systemic corticosteroids remain a cornerstone in the management of VEXAS syndrome due to their efficacy in controlling inflammatory symptoms, their long-term use is limited by significant side effects and the potential for steroid dependence. This underscores the need for alternative therapeutic strategies that can provide sustained disease control with a more favorable safety profile.

### 3.2. Ruxolitinib and Other JAK Inhibitors

Janus kinase inhibitors (JAKi) represent a class of targeted therapies used in the treatment of VEXAS syndrome, with prominent agents including ruxolitinib, baricitinib, upadacitinib, and tofacitinib [24,30,31]. These inhibitors modulate the JAK-STAT signaling pathway, which is integral to inflammation and immune regulation [31]. In a systematic review by Boyadzhieva et al. [30], 33 of 116 patients with VEXAS syndrome received treatment with JAK inhibitors, specifically ruxolitinib, tofacitinib, baricitinib, and upadacitinib. Among these patients, clinical outcomes were categorized into three groups: 11 patients achieved a full clinical response, 9 exhibited partial improvement, and 13 showed no response. Ruxolitinib, in particular, has shown promising results due to its selective inhibition of JAK1 and JAK2, which are crucial mediators of inflammatory signaling. Its adaptable dosing range also allows for tailored adjustments to meet individual patient needs [31]. Furthermore, when ruxolitinib was administered following azacitidine treatment, with corticosteroid dosing unchanged, significant improvement in cutaneous lesions was observed in VEXAS patients [17], suggesting its potential as a suitable agent for managing cutaneous manifestations. However, the use of JAKi is not without risks, as adverse events were noted in treated patients. These included enterohemorrhagic Escherichia coli infection, pneumonia, urinary tract infection, venous thromboembolism, polyneuropathy, and spinocerebellar ataxia. Despite these challenges, JAKi remains a promising therapeutic option for managing the complex inflammatory and hematological features of VEXAS syndrome [30].

### 3.3. Abatacept

Abatacept is a biologic disease-modifying antirheumatic drug (DMARD) that selectively targets co-stimulatory signals essential for T-cell activation in the immune system. It operates by binding to CD80 or CD86, thereby inhibiting T-cell co-stimulation through CD28 and subsequently halting the continuous T-cell activity driving immune-mediated inflammatory diseases [32]. Although the use of abatacept in VEXAS syndrome is not extensively documented, Pathmanathan et al. presented a case where it was administered to a patient with moderate-dose corticosteroid dependence and significant systemic and hematological manifestations [29].

This patient had previously undergone multiple ineffective treatments, including golimumab, etanercept, adalimumab, methotrexate, azathioprine, and ciclosporin, yet remained corticosteroid-dependent. Upon treatment with a combination of abatacept and 5 mg of prednisone, the patient achieved a sustained clinical response lasting 30 months. Although the improvement in hematologic and systemic symptoms was not complete, it was markedly superior to that achieved with prior therapies [29].

### 3.4. IL-6 Inhibitors

Tozaki et al. identified elevated serum levels of interleukin-6 (IL-6) in patients with VEXAS syndrome, suggesting that IL-6 inhibitors like tocilizumab may offer therapeutic benefits [25]. Tozaki et al. reported a case of rapid clinical improvement after introducing intravenous tocilizumab alongside oral prednisone, highlighting its potential role in treatment [25]. While anti-IL-6 therapies can reduce corticosteroid doses and help control inflammatory symptoms in some patients with VEXAS, a significant number still experience progressive bone marrow failure [33]. This continued progression, despite IL-6 inhibition, indicates that targeting IL-6 alone may not suffice to manage the hematological aspects of VEXAS syndrome, underscoring the necessity for combination therapies or alternative approaches to address both inflammatory and hematological manifestations effectively.

A study by Kunishita et al. further suggests that combining glucocorticoids and tocilizumab may provide better outcomes for VEXAS patients with low International Prognostic Scoring System (IPSS) scores for myelodysplastic syndrome (MDS) or those exhibiting a strong inflammatory phenotype without MDS [34]. In one instance, weekly tocilizumab injections, together with corticosteroids, led to notable improvements in cutaneous and systemic symptoms, allowing the patient to become transfusion-independent, heal skin lesions, and discontinue corticosteroid therapy [35]. These findings imply that IL-6 inhibitors may be particularly beneficial for patients with predominant inflammatory symptoms and less severe hematological involvement.

However, a systematic review involving fifteen patients treated with tocilizumab found that only three achieved a clinical response. Adverse events reported included herpes zoster infection, Pneumocystis jirovecii pneumonia, nocardia infection, severe pancytopenia, neutropenia, and perforations of the ileum and jejunum [30].The effectiveness of other IL-6 inhibitors, such as siltuximab and sarilumab, remains limited in the current literature. Siltuximab, when combined with 10 mg of corticosteroids, yielded a partial response over a short follow-up period of two months. Sarilumab also produced a partial response with corticosteroid reduction; however, treatment was discontinued due to injection site reactions. After discontinuation, the patient experienced a flare-up of auricular inflammation, episcleritis, arthralgia, and skin lesions, indicating a relapse of symptoms [36]. Careful patient selection and vigilant monitoring for infections and gastrointestinal complications are essential when considering this treatment option, as adverse reactions leading to discontinuation can result in rapid disease relapse.

### 3.5. Different TNF Inhibitors: Infliximab, Adalimumab and Etanercept

Tumor necrosis factor-alpha (TNF-α) inhibitors, such as infliximab, adalimumab, and etanercept, have been used as steroid-sparing agents in the management of VEXAS syndrome, though their efficacy appears to be limited. In a systematic review, four patients received TNF inhibitors alongside corticosteroids: one with etanercept, one with infliximab, one with infliximab combined with methotrexate, and one with adalimumab combined with methotrexate. All patients showed no clinical response, except for the one treated with adalimumab and methotrexate, who developed Pneumocystis jirovecii pneumonia as an adverse event [30]. Furthermore, in a Canadian cohort, two patients were treated with infliximab and etanercept in combination with other medications. Infliximab demonstrated a lack of efficacy and was discontinued due to the incidental discovery of rectal cancer. Conversely, etanercept resulted in decreased inflammatory symptoms and allowed for corticosteroid dose reduction [36]. In the Spanish cohort involving thirty patients, three received anti-TNF agents and none exhibited a positive response [11]. Adalimumab was transiently effective in three patients, with a median time to the next treatment of 3.4 months [17].

These findings suggest that TNF-α inhibitors may have limited efficacy in VEXAS syndrome and could be associated with significant adverse effects. The lack of response in most cases raises questions about the role of TNF-α in the pathophysiology of VEXAS syndrome, indicating that TNF-α may not be a primary driver of the inflammatory process in this condition.

### 3.6. Stem Cell Transplantation

Allogeneic hematopoietic stem cell transplantation (alloHCT) has shown promising efficacy as a potential cure for patients with severe, refractory VEXAS syndrome, particularly those with hematologic involvement such as MDS, offering hope where conventional therapies have failed. Published reports indicate that patients undergoing AlloHCT following the failure of prior treatments often achieve durable remission, with normalization of bone marrow morphology and inflammatory markers in many cases [37,38,39,40]. Additionally, long-term follow-up has demonstrated that a significant number of patients remain relapse-free and can discontinue immunosuppressive therapy, supporting alloHCT’s potential as a curative approach in VEXAS syndrome [39,40].

Despite the promising outcomes, alloHCT for VEXAS syndrome is associated with significant post-transplant complications, particularly infections and graft-vs-host-disease (GVHD), which necessitate vigilant monitoring and comprehensive supportive care [37,39,40]. All patients require prophylaxis against GVHD with immunosuppressive regimens, including agents such as cyclophosphamide, tacrolimus, mycophenolate mofetil, cyclosporine, and methotrexate. While these regimens are essential, they also introduce their own risks and side effects. Reported complications have included drug-induced rashes, mucositis, mild dermal hypersensitivity, steroid withdrawal effects, lactic acidosis, encephalopathy, bacteremia, and viral complications such as BK virus-associated hemorrhagic cystitis and cytomegalovirus replication [30,37,38,39]. Notably, GVHD incidence varied among studies: Mangaonkar et al. [39] observed only mild, grade 1 acute skin GVHD in one patient, whereas Diarra et al. [37] reported GVHD in five of six patients. Furthermore, alloHCT is not universally accessible, and many patients who could potentially benefit from this therapy are deemed ineligible due to factors such as age, comorbidities, or lack of suitable donors, further complicating the treatment landscape for VEXAS syndrome. These limitations underscore the need for optimized post-transplant care protocols and broader accessibility to improve patient outcomes and mitigate associated risks.

Further research and clinical trials are necessary to optimize transplantation protocols, improve patient outcomes, and identify the most suitable candidates for alloHCT in VEXAS syndrome, considering its potential complications and side effects, as well as the availability of alternative medical treatment options. For a comprehensive overview of the published reports utilizing hematopoietic stem cell transplantation in VEXAS syndrome, refer to Table 1. 

### 3.7. Azacytidine

Azacytidine is a pyrimidine nucleoside analog of cytidine, widely used to treat various hematological disorders, including MDS, which is strongly associated with VEXAS syndrome. Its mechanism of action involves the inhibition of DNA methyltransferase, reducing methylation in newly synthesized DNA. This demethylating effect leads to the activation of previously silenced genes, thereby promoting tumor-suppressing functions and inducing cellular differentiation [42]. Azacytidine is considered one of the primary agents in the treatment of high-risk MDS. Additionally, it has shown efficacy in treating certain autoinflammatory diseases (AID) associated with MDS. In a retrospective study evaluating azacytidine’s effectiveness in patients with AID and concurrent MDS, significant clinical improvement was reported in 86% of patients, underscoring its anti-inflammatory potential [43].

Building on these findings, azacytidine has demonstrated considerable promise in the management of VEXAS syndrome. Across multiple studies and case reports, azacytidine has proven effective in alleviating disease-related inflammatory symptoms, achieving transfusion independence, and reducing corticosteroid dependence in patients with VEXAS syndrome [44,45,46,47,48]. In one study, patients treated with azacytidine (75 mg/m^2^ s.c. daily for seven days in a four-week cycle) showed a remission of inflammatory symptoms and the normalization of hematologic parameters, with some achieving steroid tapering or discontinuation [44].

Data from other cohorts further emphasize azacytidine’s therapeutic benefits in VEXAS syndrome. In one cohort of four patients with both VEXAS and MDS, all achieved resolution of inflammatory symptoms within 1–3 cycles, with three patients reaching transfusion independence after 3–5 cycles [45]. Additionally, a retrospective analysis from the French VEXAS registry examined 11 patients with VEXAS and MDS and reported clinical responses in five patients over a median of 11 treatment cycles, with reduced inflammatory markers and steroid requirements [18]. Furthermore, a long-term follow-up study demonstrated the full remission of inflammatory symptoms, the normalization of blood counts, and successful steroid discontinuation over a follow-up period extending beyond 50 months, highlighting the durable benefits of azacytidine in managing VEXAS [49].

For patients with concurrent DNMT3A mutations, responses to azacytidine have been particularly favorable, suggesting that this mutation may serve as a predictive biomarker for treatment efficacy in VEXAS syndrome [44]. In one report, two VEXAS patients with DNMT3A mutations experienced complete remission of disease-related inflammatory manifestations, including skin conditions such as polychondritis and Sweet syndrome, following azacytidine therapy. These patients also demonstrated the normalization of blood values and inflammatory parameters, as well as the eradication of the UBA1-DNMT3A mutated clone. Importantly, bone marrow findings in these patients returned to a normal morphology, signifying a profound hematologic response alongside clinical remission [44]. Further supporting this effect, a report involving six patients with VEXAS syndrome, four of whom had concurrent MDS, demonstrated that azacytidine therapy led to remission in five patients. This cohort showed notable decreases in inflammatory symptoms and parameters, reductions in UBA1- and DNMT3A-mutated clones, and improved hematologic profiles, reinforcing azacytidine’s impact on disease stabilization in patients with DNMT3A mutations [46]. This correlation between DNMT3A mutations and improved response to azacytidine highlights the potential of DNMT3A as a biomarker that could guide therapeutic decisions in VEXAS syndrome. While adverse events such as hematologic toxicities have been observed [46,50], along with rare instances of pneumonitis [44], azacytidine maintains a favorable safety profile, especially relative to alternative therapies.

The consistent positive responses across these studies underscore azacytidine’s role as a viable therapeutic option for VEXAS syndrome, with potential for both clinical and molecular remission in patients unresponsive to conventional treatments. For additional details on treatment protocols, efficacy outcomes, and adverse events, refer to Table 2, which provides a comprehensive summary of published studies on azacytidine in VEXAS syndrome.

### 3.8. CHOP Therapy

Cyclophosphamide, doxorubicin hydrochloride (hydroxydaunorubicin), vincristine sulfate (Oncovin), and prednisone—collectively known as CHOP therapy—is a chemotherapy regimen conventionally used in the treatment of non-Hodgkin’s lymphoma [52]. The effectiveness of CHOP regimen in the treatment of VEXAS syndrome was demonstrated in one report where the patient received a single course CHOP therapy, then had a significant improvement in his clinical condition [53]. Although the patient improved significantly after CHOP therapy, he had multiple overlapping illnesses; therefore, a larger scale study should be conducted in the future to explore its effectiveness in the treatment of VEXAS syndrome. Cyclophosphamide and methylprednisolone were shown to be effective in the induction of remission in two patients reported in a case series. Although this combination resulted in remission, a reduction in steroids dose was not possible, making it inappropriate for maintenance [3].

### 3.9. Fludarabine and Cyclophosphamide

Fludarabine and cyclophosphamide are commonly used as conditioning agents before peripheral blood stem cell transplantation (PBSCT) to facilitate a successful transplant [54]. In a case report, a patient with VEXAS syndrome and MDS received two doses of fludarabine (30 mg/m^2^) and cyclophosphamide (14.5 mg/kg) prior to HLA-haploidentical PBSCT. However, the transplantation was delayed due to a pulmonary fungal infection, which required treatment before proceeding. Remarkably, following infection resolution and prior to PBSCT, the patient experienced marked improvement in systemic and hematologic manifestations, including the resolution of cytopenia [55]. This observation suggests that fludarabine and cyclophosphamide may possess intrinsic therapeutic effects in VEXAS syndrome beyond their role in transplant conditioning.

### 3.10. Rituximab

Rituximab is an anti CD20 monoclonal antibody that targets a CD20 protein found on B-cells leading to the destruction of B-cells and reduction in antibody synthesis by plasma cells. Although the use of rituximab for VEXAS syndrome was reported at least in 14 cases, only 3 cases had ineffective response and another reporting recurrence of inflammation [30]. A case series described one VEXAS patient treated with rituximab infusion yearly for 3 years who experienced complete remission and discontinued steroids after therapy. However, in the same series another two patients received Rituximab in addition to other agents with no improvement in clinical status [3]. The use of rituximab in the treatment of VEXAS syndrome appears to be promising; however, further studies should be conducted to ensure its safety and efficacy.

### 3.11. Intravenous Immunoglobulins (IVIG)

IVIG therapy possesses an immunomodulatory potential through acting upon various complex mechanisms in autoimmune, immune-mediated, and inflammatory diseases. This is mainly by interfering with antigen presentation, interacting with the complement system, cytokines, chemokines, and modulating natural killer cells, T and B lymphocytes effector functions [56]. In VEXAS syndrome, the involvement of innate immunity possibly predicts a favorable response to IVIG infusion, as reported by Magnol et al. [57].

The case reported [57], was initially diagnosed with spondyloarthropathy (SpA) followed by VEXAS syndrome, for which he was treated with a combination of IVIG and anti-IL17 therapy. This resulted in a maintained response with diminished chondritis, uveitis, IBD or SpA flares, and allowed for a significant steroids dose reduction. In addition, one case series reported a novel finding, which is that treating patients who have co-existing plasma–cell myeloma with plasma–cell-directed therapy (including high dose IVIG) showed marked improvement in the inflammatory response [4].

### 3.12. Interleukin-1 (IL-1) Inhibitors: Anakinra and Canakinumab

Another drug category that targets the inflammatory cytokines and can possibly be effective for VEXAS are the IL-1 inhibitors. Anakinra has proven its short-term efficacy in reducing inflammation and relieving pain, with some inter-individual variation [28]. In one study that involved twelve VEXAS patients [3], Anakinra sustained remission for at least 1.5–2 years in two patients, whereas the effect was absent in some others. In four patients, a severe local reaction at injection site (redness, swelling and pain) developed, which made efficacy evaluation not possible. Those patients were switched to Canakinumab, which is a selective IL-1β inhibitor, and some of them showed a good response [3]. It has been concluded by various studies that VEXAS patients would develop a more pronounced local inflammatory skin reaction to Anakinra subcutaneous administration compared to other patients, which could potentially suggest a diagnostic clue [3,58,59]. This could be avoided by intravenous Anakinra and desensitization therapy [60]. In other words, most patients have at least a partial response to Anakinra, but with inevitable disease recurrence or intolerance that eventually leads to treatment discontinuation [3,11,59]. Furthermore, Campochiaro et al. reported, in another cohort study, that introducing cyclosporin A in combination with Anakinra has diminished the appearance of adverse cutaneous reactions, resulted in the marked improvement of systemic manifestations in all patients, and allowed for safe steroids therapy tapering [61].

### 3.13. Conventional DMARDs: Methotrexate, Mycophenolate, Azathioprine, Cyclophosphamide

Conventional disease modifying anti-rheumatic drugs (DMARDs) are usually administered to treat inflammatory and rheumatological manifestations of VEXAS syndrome. However, they are mostly efficacious temporarily and may partially relieve symptoms [17,28]. An Italian case series that involved patients with vasculitis and coexisting features of VEXAS syndrome confirms that VEXAS patients are resistant to multiple DMARDs, including azathioprine and cyclophosphamide [12]. In the Spanish cohort mentioned previously [11], methotrexate, cyclophosphamide, mycophenolate, and azathioprine were all administered separately to different patients in attempt to decrease the corticosteroid dose, and none of them has resulted in complete response, yet a partial response was observed in less than 50%, with the rest showing a negative response. Additionally, the results of the Canadian cohort show that methotrexate and mycophenolate lacked efficacy when given to VEXAS patients, and methotrexate specifically led to intolerable side effects (abdominal pain, nausea, and macrocytic anemia), and therefore, both were discontinued. On the other hand, Cyclophosphamide resulted in a partial response but was discontinued due to cytopenia. Azathioprine was variable in its efficacy, showing a response in some patients, which was evident by a reduction in the CRP level and a good control of symptoms while tapering prednisolone, but had a lack of efficacy in others and severe side effects in some of the patients, including fever, anemia, and oral ulcers, due to which it was discontinued [36]. Pathmanathan et al. reported that attempts of methotrexate, azathioprine, and other immunosuppressive agents all failed in a patient with significant hematological disturbances (myelodysplastic syndrome and cytopenia) and major systemic manifestations, which made the patient steroid-dependent [29]. Regarding methotrexate, the median time to the next treatment was 7.4 months, as noted by Bourbon et al. [17]. Overall, the limited and variable responses to conventional DMARDs highlight the need for alternative therapeutic strategies in VEXAS syndrome.

In summary, a range of potential steroid-sparing treatments have been investigated for VEXAS syndrome, aimed at achieving both inflammatory control and hematologic stability. For a detailed summary of these therapeutic options, refer to Table 3.

## 4. Discussion and Conclusions

The heterogeneity of VEXAS syndrome necessitates a personalized approach to treatment. An in-depth understanding of specific UBA1 mutations and their influence on disease expression could inform therapy selection and improve prognostication. There is a critical need for large-scale, multicenter clinical trials to systematically assess the efficacy and safety of promising therapies. Such trials should aim to establish standardized treatment protocols, optimal dosing regimens, and monitoring strategies to equip clinicians with evidence-based guidelines for managing this complex condition. Additionally, exploring combination therapies that address both the inflammatory and hematologic aspects of VEXAS syndrome may provide synergistic therapeutic benefits.

Research should prioritize uncovering the molecular mechanisms that drive the inflammatory and hematologic manifestations of VEXAS syndrome. This includes investigating the role of the ubiquitin–proteasome system in immune regulation and identifying biomarkers that may predict treatment response. Collaboration among rheumatologists, hematologists, immunologists, and researchers is essential to developing comprehensive care strategies. Establishing international registries and standardized protocols will improve data collection and deepen our understanding of the disease. Ultimately, advancing patient care in VEXAS syndrome will rely on sustained research efforts to unravel its underlying mechanisms and develop targeted, effective therapies. Through collaborative, multidisciplinary approaches, we can transform VEXAS syndrome from a fatal condition to a manageable disease with improved outcomes for patients.

## Figures and Tables

**Table 1:** Summary of published reports on allogeneic hematopoietic stem cell transplantation (alloHCT) outcomes in VEXAS syndrome.

Author, Year [Ref.]	Type of Study	*n*	Sex M (%)	Population/Median Follow-Up (FU)	Therapeutic Protocol	Prior IST *n* (%)	Main Efficacy Outcomes	**Serious Adverse Events**
Diarra et al., 2022 [37]	Case series	6	6 (100%)	VEXAS syndrome was observed in six cases, with four cases associated with MDS alone (4/6), one case presenting with both myelofibrosis and MDS (1/6), and one case with myelofibrosis alone (1/6)/FU: 24.8 months	All patients underwent AHCT following lack of response to multiple therapeutic interventions.	6 (100%)	Complete response (5/6); death before evaluation (1/6)	Infectious complications (4/6), GVHD (5/6), death before evaluation (1/6)
van Leeuwen-Kerkhoff et al., 2022 [38]	Case report	1	1/1 (100%)	VEXAS syndrome/FU: 9 months	Induction therapy: myeloablative regimen comprising thiotepa, fludarabine, anti-thymocyte globulin (ATG-Fresenius), and melphalan. Post-transplant, the patient received GVHD prophylaxis.	1/1 (100%)	Hematologic recovery was observed 10 days post-transplant. At six weeks post-transplant, 100% donor chimerism was achieved. Mycophenolate was discontinued after one month, and prednisone was tapered off at 3.5 months post-transplant, with no reintroduction by the 9-month follow-up.	CMV colitis and viral enteritis were observed in this patient 15 days post-transplant.
Al-Hakim et al., 2022 [41]	Case series	4	4 (100%)	VEXAS syndrome was observed in four patients, with two of these cases associated with MDS/FU: 13.5 months	Three patients underwent AHCT due to severe, poorly controlled illness. In one case, initially suspected to be MDS alone, VEXAS syndrome was identified only after transplantation.	4 (100%)	The first patient experienced rapid deterioration post-transplant due to infections complicated by sepsis, multiorgan failure, and cardiac arrest, and subsequently died. The second patient initially improved but later had infectious and immune complications, resulting in a Karnofsky performance score of 40. The third patient achieved an initial remission, with inflammatory illness in remission; however, neurological decline and infection ultimately led to death. The fourth patient tolerated the transplant well and is currently in remission.	The first patient developed sepsis, multiorgan failure, and cardiac arrest. The second patient experienced inflammatory encephalitis, HLH, EBV reactivation, severe peripheral muscle wasting, GVHD, recurrent bacterial respiratory and urinary infections, and hypogammaglobulinemia. The third patient suffered from complete paraplegia, vision loss, infection, and ultimately death. The fourth patient exhibited minimal GVHD.
Mangaonkar et al., 2023 [39]	Case series	5	5 (100%)	VEXAS syndrome was observed in three cases of refractory inflammatory symptoms (3/5), one case with transfusion-dependent anemia and polychondritis (1/5), and one case with MDS (1/5)/FU: 9.6 months	All patients underwent AHCT following lack of response to multiple therapeutic interventions.	5 (100%)	All patients demonstrated improvement in VEXAS-related inflammatory manifestations, accompanied by normalization of bone marrow morphology and inflammatory marker levels.	Mucositis (2/5), bacteremia (1/5), encephalopathy/steroid withdrawal (1/5), drug-induced rash (1/5), delayed count recovery (1/5), lactic acidosis (1/5), weakness (1/5), difficile induced diarrhea (1/5), E-coli infection (1/5), GVHD (1/5), mild dermal hypersensitivity reaction (1/5).
Gurnari et al., 2024 [40]	Case series	19	19 (100%)	VEXAS syndrome was observed alongside myelodysplastic syndromes (MDS) in thirteen cases, with five cases presenting with autoinflammatory manifestations, and one case associated with a myeloproliferative neoplasm (MPN)/FU: 14 months	All patients underwent AHCT following lack of response to multiple therapeutic interventions.	19 (100%)	All patients remained relapse-free from VEXAS syndrome, and in cases with over one year of follow-up, all demonstrated the successful discontinuation of immunosuppressive therapy post-transplant. However, four patients died due to complications, including bacterial infections in three cases and CNS toxicity in one case.	Primary graft failure (18/19), acute GVHD (11/19), grade 2 to 4 GVHD (5/19), chronic GVHD (4/19), bacterial infection and severe multiple refractory chronic GVHD (1/19).

CMV: cytomegalovirus, CNS: central nervous system, EBV: Epstein–Barr virus, FU: follow up, GVHD: graft versus host disease, HLH: hemophagocytic lymphohistiocytosis, IST: immunosuppressive therapy, MDS: myelodysplastic syndromes, VEXAS: vacuoles, E1 Enzyme, X-linked, autoinflammatory, somatic.

**Table 2:** Azacytidine in VEXAS syndrome: published reports and studies.

Author, Year [Ref.]	Type of Study	*n*	Sex M (%)	Population/Median Follow-Up (FU)	Therapeutic Protocol	Prior IST *n* (%)	Concomitant Use of Steroids *n* (%)	Main Efficacy Outcomes	Serious Adverse Events
Cordts I et al., 2022 [47]	Case report	1	1 (100%)	VEXAS syndrome and MDS FU: NA	5-AZA 75 mg/m^2^/day subcutaneously; however, the number of cycles were not specified	1 (100%)	1 (100%)	Improvement of cytopenia (1/1), resolution of inflammatory symptoms (1/1), transfusion independence (1/1), and reduction in the dose of steroids (1/1)	None recorded
Kataoka A et al., 2023 [48]	Case report	1	1 (100%)	VEXAS syndrome and MDS FU: 3 months	5-AZA 75 mg/m^2^ subcutaneous injection, once daily for 7 days in a 4-week schedule for 6 cycles	0	1 (100%)	Improvement in fever and skin rash (1/1), reduction in the dose of steroids (1/1), and arrest of cytopenia progression	None recorded
Aalbers AM et al., 2024 [46]	Case series	6	6 (100%)	VEXAS syndrome and only 4 had associated MDS. FU: median is 31 months (range 11–84 months)	5-AZA 75 mg/m^2^ subcutaneous injection, once daily for 7 days in a 4-week schedule for at least 3 cycles	6 (100%)	6 (100%)	Clinical and genetic response (5/6), cessation of steroids (4/6), and reduction in the dose of steroids the fifth patient who had a response	Neutropenia (2/6)
Sockel K et al., 2024 [49]	Case series	2	2 (100%)	VEXAS syndrome and MDS FU: 52 months	5-AZA 75 mg/m^2^ subcutaneous injection, once daily for 7 days in a 4-week schedule for 16–18 cycles	2 (100%)	2 (100%)	Resolution of inflammatory symptoms (2/2), resolution of cytopenia (2/2), and cessation of steroids (2/2)	None recorded
Pereira da Costa R et al., 2024 [51]	Case report	1	1 (100%)	VEXAS syndrome without MDS FU: 10 months since the start of 5-AZA	5-AZA 75 mg/m^2^ subcutaneous injection, once daily for 7 days in a 4-week schedule for 9 cycles	1 (100%)	1 (100%)	Clinical and molecular remission (1/1), transfusion independence (1/1), and a reduction in the dose of steroids	None recorded
Raaijmakers MHGP et al., 2021 [44]	Case series	3	3 (100%)	VEXAS syndrome FU: NA	The first patient received a total of 8 cycles of 5-AZA 75 mg/m^2^ s.c. QD for 7 d in a 4-week schedule, the second and third patients received the same dose of 5-AZA for only 3 cycles.	3 (100%)	3 (100%)	First patient: Complete disappearance of inflammatory symptoms, loss of transfusion dependency, weaning of steroids and normalization of blood values. Second patient: Normalization of bone marrow findings, remission of inflammatory symptoms and reduction in steroid dose. Third patient: no reduction in disease burden	Interstitial pneumonitis in the third patient
Trikha R et al., 2024 [45]	Case series	4	4 (100%)	VEXAS syndrome with MDS FU: NA	All received 5-AZA 75 mg/m^2^ over 7 days in a 28-day cycle for at least 6 cycles	4 (100%)	4 (100%)	Resolution of all inflammatory symptoms (4/4), discontinuation of steroids (2/4), and reduction in the dose of steroids (2/4). Disappearance of transfusion dependence in all three patients requiring transfusion	None recorded
Bourbon E, 2021 [17]	Case series	11	11 (100%)	VXAS syndrome and only 5 of them had an associated MDS. FU: 40 months	Only 4 patients received 5-AZA (dose is not mentioned) for a median of 14.8 months	Not mentioned	4 (100%)	Patients who received azacytidine had Improvement of inflammatory manifestations and longest time to receive next treatment with steroids compared to other agents	None recorded
Comont T et al., 2022 [18]	Retrospective study	11	11 (100%)	VXAS syndrome with MDS FU: 12–75 months	5-AZA 75 mg/m^2^ per day for 5–7 days for a median of 11 cycles	9 (81.8%)	10 (90.9%)	Major clinical response (2/11), minor clinical response (3/11), no response (6/11), withdrawal of steroid (1/11), reduction in the dose of steroid (4/11), and reduction in CRP level (5/11); three patients with MDS achieved erythroid and platelets response	Pneumocystis infection (1/11), severe colitis (1/11), and bacterial pneumonia (1/11)
Mekinian A et al., 2022 [50]	Prospective trial	29	20 (69%)	MDS/CMML and SAID, only 12 had associated VEXAS syndrome. FU: 24 months	5-AZA 75 mg/m^2^ daily for 7 days every 4 weeks for at least six cycles	All patients with VEXAS	All patients with VEXAS	A total of 9/12 VEXAS patient achieved SAID and hematological response and a reduction in the dose of steroid	Hematological toxicities and infectious complications otherwise unspecified

MDS: myelodysplastic syndromes; CMML: chronic myelomonocytic leukemia; FU: follow up; VEXAS: vacuoles; E1 enzyme; X-linked, autoinflammatory, somatic; 5-AZA: azacytidine;: systemic autoimmune and inflammatory disorders.; NA: Not available; QD: Once daily; CRP: C-reactive protein.

**Table 3:** Summary of VEXAS syndrome potential steroid-sparing agents.

Medication	Main Outcome on Anti-Inflammatory Arm	Main Outcome on Hematological Arm	Serious Adverse Events and Potential Limitations	Potential Future Considerations
JAKi	Significant clinical improvement with symptom resolution and reduced steroid dose and dependency in many cases, particularly with ruxolitinib [12,17,31,62,63,64].	Some patients showed improved hemoglobin and RBC transfusion independence, while others failed to achieve independence and experienced MDS progression [12,31,65].	Infection, cytopenia, and thrombosis [31,63].	JAK inhibitors may be a highly promising class of medications for VEXAS, showing effectiveness particularly in managing inflammatory symptoms. However, their hematological benefits remain mixed, and potential infection risks warrant careful monitoring.
IL-6 inhibitors	IL-6 inhibitors, especially tocilizumab, have demonstrated clinical improvement, symptom resolution, and steroid-sparing benefits in many VEXAS patients, particularly those with dominant inflammatory symptoms [3,25,34,35].	While effective for inflammatory control, IL-6 inhibitors alone often do not prevent progressive bone marrow failure, indicating limited impact on hematologic manifestations in VEXAS. [33,34]	Cytopenia and infections [30,36,63].	Alongside JAK inhibitors, IL-6 inhibitors may be a first-line option for patients with a predominantly inflammatory presentation. However, combination therapy may be essential for those with significant hematological involvement.
Azacytidine	Improvement or complete resolution of inflammatory symptoms was observed in most studies, alongside reduction in steroid dependency [17,44,45,46,47,49].	associated with loss of transfusion dependency [44,45,47], Improvement in cytopenia [44,47,48,49], and normalization of bone marrow findings [44].	infection [18,44].	The single most promising agent for the treatment of hematological manifestations associated with VEXAS, with good outcomes in both inflammatory and hematological aspects of the disease
HSCT	Complete response and resolution of all inflammatory and hematological aspects of the disease in uncomplicated cases [30,37,38,39].	Complete response and resolution of all inflammatory and hematological aspects of the disease in uncomplicated cases [30,37,38,39].	GVHD and infectious complications [30,37,38,39].	While it offers a potential cure for VEXAS syndrome, its application is significantly restricted by the limited eligibility of patients. Furthermore, HSCT carries substantial risks, including GVHD and a high likelihood of complications if not meticulously executed.
Abatacept	In a single case, abatacept given in combination with methotrexate and leflunomide led to a sustained clinical response for 30 months, with significant reduction in steroid dose, and effective control of inflammatory symptoms [29].	In a single case, partial hematological improvement was observed [29].	Limited data on adverse events specifically related to VEXAS patients.	Larger cohorts are needed to confirm abatacept’s efficacy in VEXAS, including its potential as monotherapy or in optimal combinations for both inflammatory and hematologic control.
TNF-α inhibitors	Poor or minimal clinical response in most studies [11,17,30,36,63].	Poor or minimal clinical response in most studies [11,17,30,36,63].	Infection and thrombosis were the most prominent adverse events [30,63].	A poor candidate for treatment of VEXAS syndrome.
DMARDS (methotrexate, mycophenolate, azathioprine, cyclophosphamide)	Poor or minimal clinical response in most studies [11,12,29,30,36].	Poor or minimal clinical response in most studies [11,12,29,36].	Anemia and cytopenia [36].	A poor candidate for treatment of VEXAS syndrome.
Fludarabine and cyclophosphamide	Complete resolution of systemic manifestations of VEXAS and associated MDS in a single study [55].	Remarkable improvement in cytopenia [55]	Neutropenic fever, opportunistic infection [55].	Fludarabine and cyclophosphamide may possess an intrinsic therapeutic benefit in VEXAS syndrome beyond their role in conditioning prior to HSCT.
IVIG	Marked improvement in the inflammatory response, particularly with co-existing spondyloarthritis and plasma cell myeloma in some patients [4,57].	No data to suggest outcome.	Worsening of pulmonary complications when co-administered with mycophenolate and mofetil [12].	Larger studies may be needed to confirm the efficacy of IVIG, as a monotherapy, in the treatment of VEXAS, particularly considering the hematological outcome.
anti-CD20	Interindividual variation in treatment efficacy (good clinical and biochemical response in some patients with complete remission, whereas others developed relapse in inflammatory manifestations) [3,30,66,67,68].	One case reported an unsuccessful attempt in treating anemia and thrombocytopenia with Rituximab accompanied by IVIG [41].	Opportunistic infections [67].	The use of rituximab in the treatment of VEXAS syndrome appears to be promising, especially in patients with plasma cell dyscrasias or hematologic malignancies. However, further studies should be conducted to ensure its safety and efficacy.
IL-1 inhibitors	IL-1 blockers (anakinra and canakinumab) can be helpful in controlling the syndrome’s inflammatory and cutaneous manifestations, with some inter-individual variation [3,58,59,61,63,69].	No data to suggest outcome.	Severe skin reactions, neutropenia [3,58,59,61,63].	Most patients have at least a partial response to Anakinra, but with inevitable disease recurrence or intolerance that eventually leads to treatment discontinuation. Perhaps further clinical trials and long-term observation are needed to confirm its safety and efficacy.
CHOP	Significant clinical improvement was demonstrated by one report after a single course of the CHOP regimen, but it was not used as a maintenance therapy [3,53].	Significant clinical improvement was demonstrated by one report after a single course of the CHOP regimen, but it was not used as a maintenance therapy [3,53].	Limited data on adverse events specifically related to VEXAS patients.	A larger scale study should be conducted in the future to explore its effectiveness in the treatment of VEXAS syndrome.

JAKi: Janus kinase inhibitor, RBC: red blood cell, MDS: myelodysplastic syndrome, VEXAS: vacuoles, E1 enzyme, X-linked, autoinflammatory, somatic syndrome, IL: interleukin, HSCT: hematopoietic stem cell transplantation, GVHD: graft-versus-host disease, IVIG: intravenous immunoglobulin, CHOP: cyclophosphamide, hydroxydaunorubicin, oncovin, prednisone, DMARDs: disease-modifying anti-rheumatic drugs, TNF: tumor necrosis factor.
